# Impact of Tacrolimus Daily Dose Limitation in Renal Transplant Recipients Expressing CYP3A5: A Retrospective Study

**DOI:** 10.3390/jpm11101002

**Published:** 2021-10-02

**Authors:** Rémi Lenain, Mehdi Maanaoui, Aghilès Hamroun, Romain Larrue, Cynthia Van Der Hauwaert, Jean-Baptiste Gibier, Viviane Gnemmi, Sébastien Gomis, Myriam Labalette, Franck Broly, Benjamin Hennart, Nicolas Pottier, Marc Hazzan, Christelle Cauffiez, François Glowacki

**Affiliations:** 1 CHU Lille, Service de Néphrologie, F-59000 Lille, France; Remi.LENAIN@CHRU-LILLE.FR (R.L.); mehdi.maanaoui@chru-lille.fr (M.M.); Aghiles.HAMROUN@CHRU-LILLE.FR (A.H.); Sebastien.GOMIS@chu-lille.fr (S.G.); marc.hazzan@chru-lille.fr (M.H.); francois.glowacki@chru-lille.fr (F.G.); 2 UMR9020-U1277—CANTHER—Cancer Heterogeneity, Plasticity and Resistance to Therapies, University Lille, CNRS, Inserm, CHU Lille, F-59000 Lille, France; Romain.LARRUE@CHRU-LILLE.FR (R.L.); cynthia.vanderhauwaert@inserm.fr (C.V.D.H.); nicolas.pottier@univ-lille.fr (N.P.); 3 CHU Lille, Service de Toxicologie et Génopathies, F-59000 Lille, France; Franck.BROLY@CHRU-LILLE.FR (F.B.); Benjamin.HENNART@CHRU-LILLE.FR (B.H.); 4 CHU Lille, Département de la Recherche en Santé, F-59000 Lille, France; 5 CHU Lille, Service d’Anatomo-Pathologie, F-59000 Lille, France; Jeanbaptiste.GIBIER@CHRU-LILLE.FR (J.-B.G.); Viviane.GNEMMI@CHRU-LILLE.FR (V.G.); 6 CHU de Lille, Institut D’Immunologie-HLA, F-59000 Lille, France; myriam.labalette@chru-lille.fr

**Keywords:** pharmacogenetics, polymorphism, tacrolimus, renal transplantation, therapeutic drug monitoring

## Abstract

The pharmacokinetic variability of tacrolimus can be partly explained by CYP3A5 activity. Our objective was to evaluate a tacrolimus sparing policy on renal graft outcome according to *CYP3A5* 6986A>G genetic polymorphism. This retrospective study included 1114 recipients with a median follow-up of 6.3 years. Genotyping of the 6986A>G allelic variant corresponding to CYP3A5*3 was systematically performed. One year after transplantation, tacrolimus blood trough concentration (C0) target range was 5–7 ng/mL. However, daily dose was capped to 0.10 mg/kg/day regardless of the *CYP3A5* genotype. A total 208 CYP3A5*1/- patients were included. Despite a higher daily dose, CYP3A5*1/- recipients exhibited lower C0 during follow-up (*p* < 0.01). Multivariate analysis did not show any significant influence of CYP3A5*1/- genotype (HR = 0.70, 0.46–1.07, *p* = 0.10) on patient-graft survival. Glomerular Filtration Rate (GFR) decline was significantly lower for the CYP3A5*1/- group (*p* = 0.02). The CYP3A5*1/- genotype did not significantly impact the risk of biopsy-proven acute rejection (BPAR) (HR = 1.01, 0.68–1.49, *p* = 0.97) despite significantly lower C0. Based on our experience, a strategy of tacrolimus capping is associated with a better GFR evolution in CYP3A5*1/- recipients without any significant increase of BPAR incidence. Our study raised some issues about specific therapeutic tacrolimus C0 targets for CYP3A5*1/- patients and suggests to set up randomized control studies in this specific population.

## 1. Introduction

Tacrolimus is the worldwide cornerstone of immunosuppression after kidney transplantation [1,2]. This drug displays a narrow therapeutic index and may cause numerous adverse events if plasmatic concentrations are slightly above or below the appropriate range. Indeed, underexposure to tacrolimus increases the risk of graft rejection [3] whereas overexposure is associated with nephrotoxicity [4], infection, and metabolic complications such as diabetes or dyslipidemia [5]. These adverse events may affect graft and patient survivals as well as their quality of life [6]. Therapeutic drug monitoring, which most often consists of tacrolimus through blood concentration (C0) measurements [7], is routinely used in clinical practice to optimize the balance between the risk of graft rejection and drug toxicity.

Tacrolimus pharmacokinetic is complex with a wide intra- and inter-individual variability [8]. A large part of this variability has been attributed to CYP3A5 genetic polymorphisms. The major rs776746 (6986A > G) SNP (Single Nucleotide Polymorphism) inducing a splicing defect, results in the absence of both expression and activity of the CYP3A5 protein [9]. CYP3A5 expresser recipients (harboring at least one functional CYP3A5*1 allele) usually require a higher dose of tacrolimus than CYP3A5 non-expresser recipients (CYP3A5*3/*3, homozygotes for rs776746 SNP) in order to reach the C0 target [10,11].

A large number of studies focused on the impact of CYP3A5 rs776746 SNP on clinical outcomes of kidney allograft. In particular, the meta-analysis by Rojas et al. did not find any association between CYP3A5*1/- genotype (versus CYP3A5*3/*3) and biopsy proven acute graft rejection (BPAR) and also highlighted conflicting results related to chronic nephrotoxicity [12]. Long-term patient and graft survival can be viewed as a surrogate endpoint of tacrolimus nephrotoxicity. Similarly, Flahault et al. did not find any association between CYP3A5 genotypes and measured glomerular filtration rate (GFR), BPAR, and long-term graft survival [13]. In this study, C0 ranged from 5 to 7 ng/mL from one year post-transplantation regardless of CYP3A5 genotype. In consequence, CYP3A5*1/*1 patients required a higher mean daily dose (12 mg/day at 1 year post transplantation) than CYP3A5*3/*3 patients (5 mg/day at 1 year post transplantation) [13]. Furthermore, a higher prevalence of chronic nephrotoxicity was found in the literature for CYP3A5 *1/- patients compared to CYP3A5*3/*3 [14].

In our transplant kidney center, in order to reduce tacrolimus toxicity beyond one year post transplantation, our standard of care for tacrolimus C0 target is between 5 and 7 ng/mL with a tacrolimus daily dose capped at 0.10 mg/kg/day (regardless of CYP3A5 genotype and C0 levels). The rationale for this policy, that has been followed for the last 12 years, was based on a higher prevalence of chronic nephrotoxicity observed in CYP3A5 *1/- patients [14].

The aim of this retrospective study was thus to assess whether tacrolimus daily dose limitation is acceptable for CYP3A5 renal transplant recipient expressers.

## 2. Materials and Methods

### 2.1. Patients and Data Collection

A total 1114 adult patients who received a single kidney transplantation between 1 January 2007 and 31 December 2017 in Lille University Hospital Center, Nephrology and Kidney Transplantation Department, France were retrospectively included in this study. All patients received initial biological induction (antithymoglobulin or anti-CD25 antibodies) and were treated by tacrolimus for more than one year after transplantation. Immunosuppressive protocol consisted in tacrolimus, mycophenolate mofetil (initially 2 g/day, thereafter tapered), and steroids (500 mg at Day 0, 250 mg at Day 1, then 20 mg/day until Day 7). Steroids were stopped at Day 8 for patients without immunological risk nor delayed graft function. The initial daily dose of tacrolimus (ADVAGRAF^®^, Astellas^®^, Chuo City, Tokyo, Japan) was 0.15 mg/kg/day. Then, the dose was adjusted to reach C0 between 10 and 15 ng/mL the first 3 months, 8 and 12 ng/mL within the first year, and later in a range from 5 to 7 ng/mL with tacrolimus daily dose that should not exceed 0.10 mg/kg/day regardless of CYP3A5 genotype. Liver transplants and patients treated with chronic drugs known to interfere with tacrolimus were excluded.

Data were collected from the database CRISTAL (Agence de la Biomédecine, France) and from patient personal records (CNIL agreement number 2214185). General demographic features and possible confounders for allograft failure were extracted from the database. Recipient characteristics included age, gender, weight, height, body mass index (BMI), initial kidney disease, rank of transplantation, duration of dialysis before transplantation, pre transplant immunization (anti class I or class II Human Leucocyte Antigen—HLA), type of dialysis before transplantation, and CYP3A5 genotype. Donor features included age, gender, cause of death, and type of donor (living or deceased).

### 2.2. Tacrolimus Dosage

Tacrolimus blood concentration was measured by Architect^®^ Tacrolimus immunoassay (Abbott Laboratories, Chicago, IL, USA). The tacrolimus daily dose, the trough blood concentration (C0) and the dose-adjusted ratio (C0/daily dose) were obtained for all patients.

### 2.3. CYP3A5 Genotyping

Each recipient DNA was extracted from a peripheral blood sample using the Nucleon BACC Genomic DNA Extraction Kit (GE Healthcare, Saclay, France). Genotyping of the CYP3A5 6986A>G (rs776746) SNP was performed with TaqMan allelic discrimination assays on a ABIPrism 7900HT (Applied Biosystems, Waltham, MA, USA) as previously described [15]. When patients carried at least one CYP3A5*1, genotyping of CYP3A5*6 (rs10264272) and CYP3A5*7 (rs41303343) SNPs was further determined by direct sequencing [16]. Considering the low allele frequency of CYP3A5*1 (18.7% of the whole population during the study period), and in accordance with the literature, patients carrying this variant (CYP3A5*1/*1 or CYP3A5*1/*3) were termed as “expresser” patients or CYP3A5 *1/- patients. Recipients carrying the CYP3A5*3/*3 genotype, responsible for the absence of CYP3A5 expression, were termed as “non-expresser” patients.

### 2.4. Outcomes

The main outcome was patient-graft survival, defined as the time between transplantation and the first event among return to dialysis, pre-emptive re-transplantation, and death (all cause) with a functional graft. Secondary outcomes were longitudinal changes in estimated glomerular filtration rate (eGFR) according to MDRD (Modification of Diet in Renal Disease) formula, biopsy proven acute rejection (BPAR) occurrence according to Banff 2015 classification [17] and death censored graft survival defined as the time between transplantation and the first event among return to dialysis and pre-emptive re-transplantation (death was right censored).

### 2.5. Statistical Analysis

Characteristics at time of transplantation between the two groups of interest (CYP3A5 *1/- and CYP3A5 *3/*3) were compared using Chi square test for categorical variables and Student *t*-test for continuous variables. Crude survival curves were obtained by the Kaplan Meier estimator [18] and compared using the log-rank test. Risk factors were studied by the corresponding hazard ratio (HR) using the Cox’s proportional hazard model [19]. Univariate analyses were performed in order to make a first variable selection (*p* < 0.20, two-sided). If the log-linearity assumption was not met, the variable was categorized in order to minimize the Bayesian information criterion (BIC). Characteristics known to be associated with long-term survival were selected a priori to be included in the final model even if not significant (recipient and donor age, cold ischemia time, and previous transplantation). Biopsy proven rejection was computed as a time dependent covariate in Cox model. Hazards proportionality was checked by log-minus-log survival curves plotting on both univariate and multivariate models. Intra Patient Variability (IPV) of tacrolimus exposure was evaluated according to [20].

Linear mixed model [21] estimated by Restricted Maximum Likelihood was used to compare longitudinal changes in eGFR from 1 year post transplantation according to the CYP3A5 status (as C0/tacrolimus daily dose, C0 and tacrolimus daily dose). CYP3A5 genotype was treated as a fixed effect associated with two random effects for baseline and slope values. If the variable was not normally distributed, we considered a relevant transformation. Then, we chose the best fit model of eGFR over time on the basis of BIC values. Univariate models were composed using three effects for each variable: on baseline value, slope (interaction with time) and CYP3A5 genotype. Among these parameters, those which were not significant (*p* > 0.20, two-tailed) were removed. If the association on the slope was significant, the corresponding association on baseline value was also considered. Finally, the selected significant variables were further analyzed in a multivariate linear mixed (backward selection procedure, *p* < 0.05, two-tailed). The normal distribution of random effect on intercept, random effect on slope, residuals, and homoscedasticity assumption were graphically assessed. All analyses were performed using the 3.6.0 version of the R software [22] with “nlme” and “survival” packages.

## 3. Results

### 3.1. Patients’ Characteristics

Characteristics of the 1114 included patients at time of transplantation are described in Table 1. A total 906 patients (81.3%) were CYP3A5 non-expressers (CYP3A5*3/*3) and 208 (18.7%) CYP3A5 expressers (34 CYP3A5 *1/*1 and 174 CYP3A5*1/*3). The only significant difference between the two groups was the time spent on dialysis which was higher in the CYP3A5*1/- group than in the CYP3A5*3/*3 group (2.5 years versus 2.1 years, *p* = 0.02). During follow up, 72 patients died with a functioning graft (including 64 in the CYP3A5*3/*3 group) and 118 returned to dialysis (including 101 in the CYP3A5*3/*3 group). In addition, 171 BPAR were observed, comprising 104 TCMR (T cell mediated rejection), 84 ABMR (Antibody-mediated rejection), 22 mixed ABMR/TCMR (data missing for 5 patients). Median follow up time in the cohort was 6.3 years (interquartile range: 3.89; 9.08 years).

**Figure 1:**
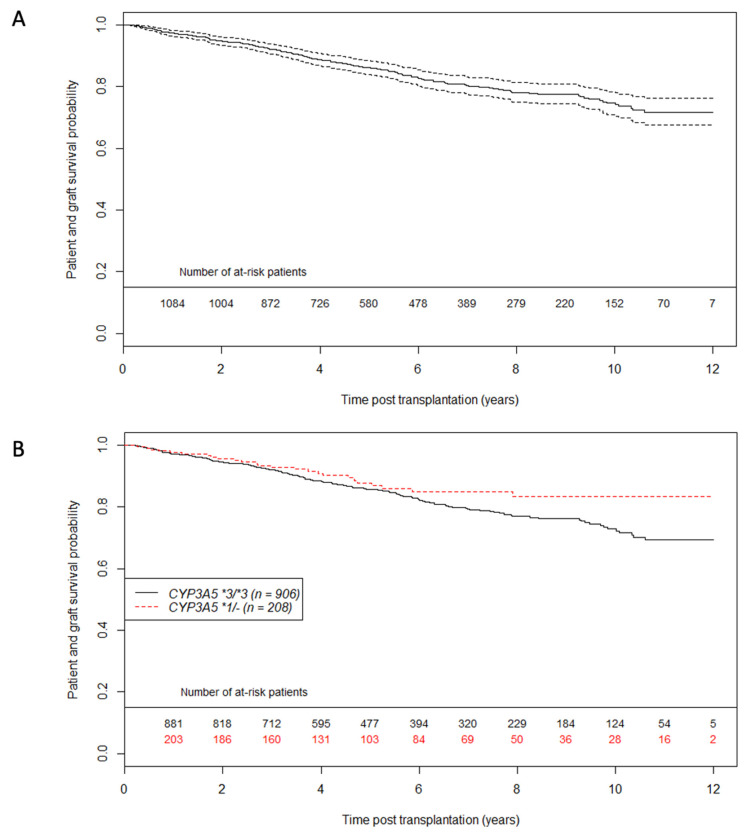
Patient graft survival unadjusted curves using the Kaplan Meier estimator (**A**) on whole population (**A**) and according to CYP3A5 genotype (**B**). Dashed lines represent 95% confidence interval. *n* = 1114 patients.

**Figure 2:**
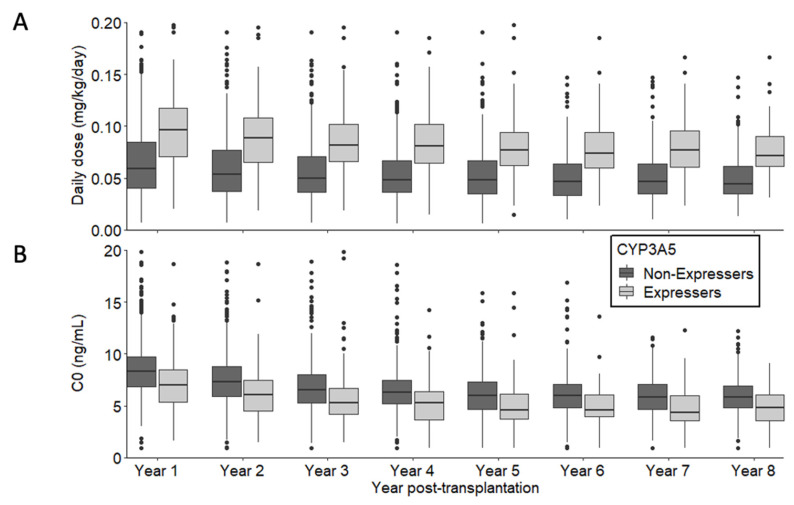
Description of tacrolimus daily dose (**A**) and C0 (**B**) from 1 year post-transplantation according to CYP3A5 expression.

Patient and graft survival curves for the entire population and according to CYP3A5 genotype are shown in Figure 1. The estimated probability of patient and graft survival in the CYP3A5*1/- group was 0.93 at 3 years post transplantation (CI95%: 0.89; 0.97) versus 0.92 in the CYP3A5*3/*3 group (CI95%: 0.90; 0.94). Graft loss etiologies were similar whatever *CYP3A5* genotype (Supplemental Table S1). Figure 2 describes tacrolimus daily dose and C0 from one year post-transplantation. As expected, daily doses were higher and C0 measures were lower in the CYP3A5 expresser group. To evaluate IPV (Intra Patient Variability) between 6 and 12 months post-transplant, coefficients of variation (CV) were calculated according to *CYP3A5* genotype. CV was higher in the CYP3A5*3/*3 group compared to CYP3A5*1/(CV = 0.201 +/− 0.200 vs. CV = 0.146 = +/− 0.150; *p* < 0.001).

### 3.2. Tacrolimus Daily dose and Trough Blood Concentration

**Figure 3:**
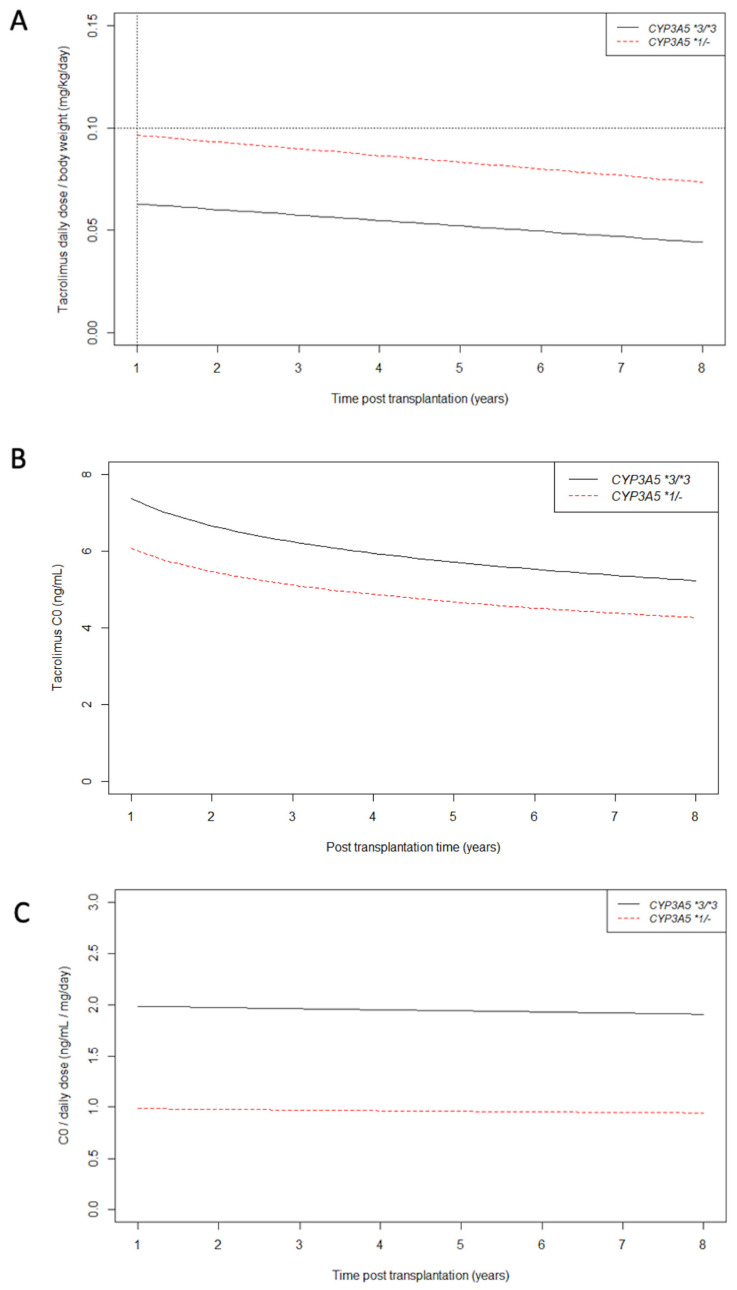
Longitudinal changes in tacrolimus daily dose/body weight (**A**), C0 (**B**) and C0/tacrolimus daily dose ratio (**C**) from 1 year post transplantation according to CYP3A5 genotype. As explained earlier, after 1 year post transplantation, the tacrolimus daily dose/body weight never exceeded 0.10 mg/kg/day regardless of CYP3A5 genotype (black dotted lines).

Linear mixed models confirmed that our clinical practice of tacrolimus daily dose capping of 0.10 mg/kg/day beyond one year post transplantation is in agreement with our care protocol (Supplemental Table S2 and Figure 3A). At one year post transplantation, the tacrolimus mean daily dose was 0.066 mg/kg/day (CI95%: 0.063; 0.068) for CYP3A5 non-expressers and 0.099 mg/kg/day (CI95%: 0.092; 0.107) for CYP3A5 expressers. Tacrolimus daily dose decreased significantly over time by 0.003 mg/kg/day for each year in average (*p* < 0.01 for time effect on slope) without any significant influence of *CYP3A5* genotype (*p* = 0.17 for CYP3A5 *1/- effect on slope).

Supplemental Table S3 and Figure 3B show the effect of the daily dose limitation of 0.10 mg/kg/day on tacrolimus trough blood concentration (C0). As expected, tacrolimus C0 measures were significantly lower in the CYP3A5 expresser group than in the non-expresser group (*p* < 0.01 for CYP3A5 *1/- effect on baseline). At 5 years post-transplantation, mean tacrolimus C0 was 5.72 ng/mL (CI95%: 5.56; 5.89) for CYP3A5 non-expressers, and 4.66 ng/mL (CI95%: 3.96; 5.36) for CYP3A5 expressers. For example, at 5 years post transplantation, 68% of CYP3A5 expressers’ C0 were lower than 5 ng/mL versus 30% for CYP3A5 non-expressers.

C0/daily dose mean ratio remained stable over time regardless of *CYP3A5* genotype (*p* = 0.22 and *p* = 0.81 for time effect and CYP3A5 effect on slope respectively) (Supplemental Table S4 and Figure 3C). As expected, the C0/daily dose mean ratio was higher in the CYP3A5 non-expresser group than in the CYP3A5 expressers group (2.00 [CI95% 1.90; 2.09] versus 0.99 [CI95% 0.79; 1.19] respectively, *p* < 0.01). The year of transplantation had no significant effect on baseline or slope values of C0/daily dose ratio (data not shown) which supports the consistency of our care protocol over the 10 years of this study.

### 3.3. Primary Outcome: Patient—Graft Survival Analysis

The multivariate analysis is shown in Table 2. The adjusted HR of death or graft failure for CYP3A5 expressers versus CYP3A5 non-expressers was 0.70 (CI95%: 0.46; 1.07, *p*-value = 0.10). We did not observe any significant association between CYP3A5 genotype and patient-graft survival in this cohort. However, we observed a trend towards a protective effect of CYP3A5 expression on graft loss. Moreover, concerning death censored graft survival (Supplemental Figure S1 and Supplemental Table S5), we did not find any significant influence of CYP3A5 genotype (HR = 0.73, CI95% 0.43; 1.23, *p =* 0.23). Concerning the graft outcomes, we found a significant association between intra patient variability (IPV) of tacrolimus and patient-graft survival (HR 1.12 for an increase of 10%; 95% CI 1.06–1.18; *p* < 0.001).

### 3.4. Secondary Outcomes: eGFR Evolution and BPAR Occurrence Analysis

**Figure 4:**
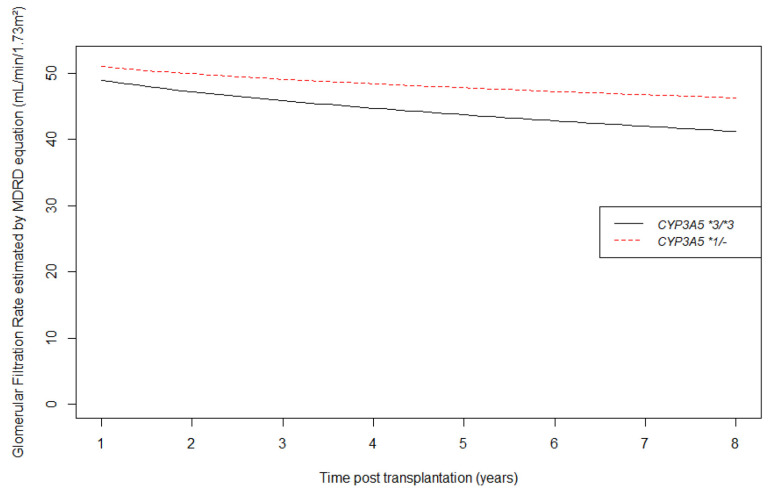
Longitudinal changes in estimated glomerular filtration rate by MDRD equation (mL/min/1.73 m^2^) from 1 year post transplantation according to CYP3A5 genotype.

Concerning eGFR, we found a better modelization using a square root transformation of time according to BIC values. Crude eGFR curves are shown in Figure 4. These crude slopes tended to be steeper in CYP3A5 non-expressers than in CYP3A5 expressers. Table 3 shows longitudinal changes by square root time unit in eGFR from one year post transplantation. *CYP3A5* genotype was not associated with one year eGFR (p = 0.64 for intercept) in the multivariate analysis, but had a significant influence on eGFR mean decrease over time (CYP3A5 expresser versus non-expresser on slope = 2.57 mL/min/1.73m^2^ per square root time unit, CI95% 0.38; 4.75, *p =* 0.02). For example, at 5 years after transplantation, a CYP3A5 non-expresser’s mean eGFR was 5.14 mL/min/1.73m^2^ lower than a CYP3A5 expresser patient, after adjustment for all potential confounders.

**Figure 5:**
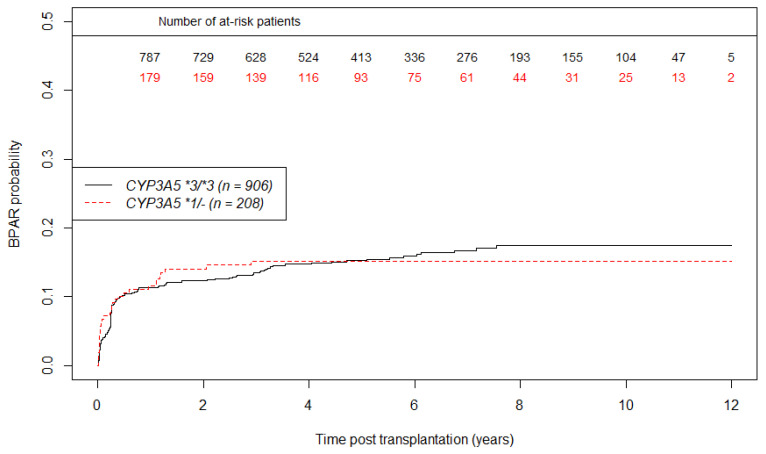
Unadjusted curves of biopsy proven acute rejection incidence using the Kaplan Meier estimator according to CYP3A5 genotype. (n = 1114 patients).

Concerning BPAR, we observed 140 graft rejection in the CYP3A5*3/*3 group versus 31 in CYP3A5 *1/- group during the follow up. Curves of BPAR incidence according to CYP3A5 status are shown in Figure 5. At one-year post transplantation, the estimated probability of BPAR occurrence is 11.6% (CI95% 6.6%; 16.5%) in the CYP3A5 expresser group, and 11.3% (CI95% 9%; 13.6%) in the CYP3A5 non-expresser group. We did not find any significant association between *CYP3A5* genotype and BPAR (HR = 1.01; CI95% 0.68; 1.49, *p =* 0.97) as shown in the multivariate analysis of BPAR in Table 4.

## 4. Discussion

By capping tacrolimus daily dose to 0.10 mg/kg/day and therefore accepting significantly lower C0 levels, our tacrolimus sparing policy was associated with a better graft function in CYP3A5 expresser patients. Furthermore, in the multivariate analysis, the incidence of BPAR in CYP3A5 expressers population did not significantly increase. Nevertheless, we did not find any significant association between CYP3A5 genotype and patient-graft survival in this context of tacrolimus sparing policy, even if there was a trend in favor of CYP3A5 expressers.

This cohort is among the largest cohorts published on the association between CYP3A5 genetic polymorphisms and long-term kidney transplantation outcomes. One of the key features of our kidney transplant center is the 0.10 mg/kg/day tacrolimus daily dose capping policy that had never been described before to our knowledge. This threshold mainly affects CYP3A5 expressers since C0 targets are most often obtained without exceeding the daily dose limit for CYP3A5 non-expressers. In consequence, this policy explains observed C0 differences between the CYP3A5 expressers and non-expressers. Thus, our sparing policy mainly affects CYP3A5 expressers. Concerning graft survival, this work did not show any influence of the CYP3A5 genotype. This finding is consistent with the available literature [13,23]. In this study, we considered graft survival as a proxy of tacrolimus chronic nephrotoxicity [4]. Indeed, tacrolimus toxicity is difficult to assess because of nonspecific histological findings and no available biomarker which could partly explain the discrepancies between past studies [12]. Nevertheless, while we did not find any significant difference on graft survival according to CYP3A5 genotype, it is important to note a trend towards a protective effect of the CYP3A5*1/- genotype. This finding should be interpreted with caution. We cannot know if it remained residual confounding after adjustment due to unobserved confounding factors or if our study was underpowered because of the small number of CYP3A5 expressers (18%). A part of the answer could lie in the eGFR analysis which showed a faster decline of graft function for CYP3A5*3/*3 patients compared to CYP3A5*1/- patients. This result is conflicting with Flahault et al. despite the same methodology, which could be explained by our daily dose capping policy [13]. The potential pitfall of a tacrolimus sparing policy is the risk of allograft rejection. Dugast et al. remind us that tacrolimus sparing is not completely risk-free even for low immunological risk patients [3]. Furthermore, the balance between risk and benefits of low C0 could be modulated by intra patient variability of tacrolimus exposure [20,24]. This point appears to be a major concern for patients with low tacrolimus exposure (C0). However, we did not find a CYP3A5 genotype influence on graft rejection.

This study has several limitations. Firstly, the sample size of CYP3A5 expressers is quite small because patients in our center are mainly Caucasian for whom the CYP3A5*3 allele is predominant [25]. Therefore, our work can suffer from a lack of power to reach the significance threshold. Secondly, all patients received the same tacrolimus sparing policy. In order to confirm the beneficial effect of the sparing policy for CYP3A5 expressers, the optimal control group would have been another cohort of CYP3A5 expressers without tacrolimus daily dose minimization. Moreover, this study design would also help to verify if the benefit observed for CYP3A5 expressers’ eGFR was not, in reality, a detrimental effect for CYP3A5 non-expressers. Thirdly, besides BPAR, de novo donor specific antibody emergence was not analyzed. Fourthly, in this retrospective study, residual confounding could remain after adjustment, in particular for ethnicity. For French regulatory issues, it was unfortunately not possible to collect this information. Finally, we did not assess in this study neither the donor genotype nor other recipient genetic polymorphisms affecting ABCB1 [15] or CYP3A4 [26] also known to potentially modify tacrolimus pharmacokinetics. A donor-recipient combined analysis could be a more precise approach for further studies and may provide a better understanding for the future. Alternatively, a whole genome approach could also be an interesting perspective that has recently emerged [27,28]. Our results need further confirmation with, for example, a randomized trial comparing capped and not-capped tacrolimus daily dose policies, or a study pooling multicenter observational data already available.

## 5. Conclusions

To conclude, this study reports long-term clinical outcomes associated with a tacrolimus sparing policy in a cohort of kidney transplant recipients according to CYP3A5 status. Even if we did not observe any association between CYP3A5 genotype and patient-graft survival, CYP3A5 expressers seem to have a better glomerular filtration rate over time than CYP3A5 non-expressers without any increased incidence of biopsy proven acute rejection.

## Figures and Tables

**Table 1:** Recipient and donor characteristics according to CYP3A5 genotype (n = 1114).

	CYP3A5 *3/*3 N = 906	CYP3A5 *1/- N = 208	*p*-Value	Available Data
Year of transplantation			0.20	1114
- 2007–2009	232 (25.6%)	40 (19.2%)		
- 2010–2012	239 (26.4%)	54 (26.0%)		
- 2013–2015	284 (31.3%)	72 (34.6%)		
- 2016–2017	151 (16.7%)	42 (20.2%)		
Recipient age (years)	52.4 (40.1;60.3)	49.9 (37.9;59.6)	0.18	1114
Recipient male	561 (61.9%)	127 (61.1%)	0.88	1114
Recipient BMI (kg/m²)	24.4 (21.4;27.6)	24.6 (22.0;27.4)	0.76	1112
Positive anti-HLA class I antibodies	169 (18.7%)	40 (19.2%)	0.93	1114
Positive anti-HLA class II antibodies	180 (20.1%)	47 (22.7%)	0.47	1101
Retransplantation	152 (16.8%)	35 (16.8%)	1.00	1114
Time spent in dialysis (years)	2.1 (1.1;3.6)	2.5 (1.3;4.6)	0.02	1111
Renal replacement therapy modality			0.14	1114
- Peritoneal dialysis	116 (12.8%)	18 (8.7%)		
- Hemodialysis	689 (76.0%)	171 (82.2%)		
- Pre-emptive transplantation	101 (11.1%)	19 (9.1%)		
Recipient blood type			0.36	1114
- A	415 (45.8%)	82 (39.4%)		
- AB	36 (4.0%)	9 (4.3%)		
- B	86 (9.5%)	25 (12.0%)		
- O	369 (40.7%)	92 (44.2%)		
Donor age (years)	52.0 (41.0;62.0)	51.0 (40.8;61.0)	0.52	1114
Donor male	537 (59.3%)	122 (58.7%)	0.93	1114
Donor BMI (kg/m²)	25.6 (22.9;28.6)	25.0 (22.5;28.6)	0.46	1114
Donor blood type			0.24	1114
- A	396 (43.7%)	75 (36.1%)		
- AB	26 (2.9%)	7 (3.4%)		
- B	78 (8.6%)	22 (10.6%)		
- O	406 (44.8%)	104 (50.0%)		
Donor vital status			0.73	1114
- Living donor	77 (8.5%)	16 (7.7%)		
- Non cerebrovascular donor death	383 (42.3%)	95 (45.7%)		
- Cerebrovascular donor death	418 (46.1%)	89 (42.8%)		
- Donor after cardiac death	28 (3.1%)	8 (3.8%)		
HLA-A-B-DR incompatibilities > 4	221 (24.4%)	65 (31.2%)	0.05	1113
Cold ischemia time (hours)	16.0 (12.0;21.0)	16.0 (12.0;20.0)	0.77	1098
Machine perfusion conservation	175 (19.4%)	37 (18.0%)	0.72	1106

Abbreviations: BMI = Body Mass Index, HLA = Human Leucocyte Antigen, BPAR = Biopsy Proven Acute Rejection. Categorical and continuous variables are expressed by count (percentage) and median value (first and third quartile) respectively.

**Table 2:** Multivariate Cox model for patient-graft survival.

	HR	CI95%	*p*-Value
CYP3A5 *1/- (versus CYP3A5 *3/*3)	0.70	(0.46; 1.07)	0.10
Recipient age > 60 years old (yes *versus* no)	2.13	(1.46; 3.12)	<0.01
Donor age > 60 years old (yes *versus* no)	1.62	(1.10; 2.37)	0.01
Male recipient (yes *versus* no)	1.38	(1.02; 1.89)	0.04
Retransplantation (yes *versus* no)	1.52	(1.02; 2.26)	0.04
Renal replacement therapy modality			
- Peritoneal dialysis	Ref.		
- Hemodialysis	1.10	(0.69; 1.75)	0.68
- Pre-emptive transplantation	0.38	(0.15; 0.97)	0.04
Time spent in dialysis (per 1 year)	1.04	(1.01; 1.07)	< 0.01
Donor vital status			
- Living donor	Ref.		
- Non cerebrovascular donor death	1.53	(0.60; 3.88)	0.37
- Cerebrovascular donor death	1.79	(0.71; 4.53)	0.22
- Donor after cardiac death	3.44	(1.10; 10.74)	0.03
Cold ischemia time (per 10 h)	1.09	(0.86; 1.38)	0.49
Occurrence of BPAR (yes *versus* no)	2.69	(1.95; 3.71)	<0.01

Abbreviations: HR = Hazard Ratio, CI95% = Confidence interval 95%, BPAR = Biopsy Proven Acute Rejection. Recipient and donor age were both categorized because of log linearity assumption violation. Occurrence of BPAR was a time dependent covariate. 22 observations were deleted due to missingness.

**Table 3:** Linear mixed model for estimated glomerular filtration rate by MDRD equation (mL/min/1.73 m^2^) from 1 year post transplantation.

	Association with 1-year Egfr (Baseline Effect)			Association with eGFR Evolution from 1 year Post Transplantation (Slope Effect)		
	Coefficients	CI95%	*p*-Value	Coefficients	CI95%	*p*-Value
Referential value	99.95	(89.49; 110.41)	<0.01	−10.40	(−15.88; −4.93)	<0.01
CYP3A5 *1/- (ref: CYP3A5 *3/*3)	−0.87	(−4.56; 2.82)	0.64	2.57	(0.38; 4.75)	0.02
Recipient age (years)	−0.10	(−0.24; 0.03)	0.15	0.08	(0.02; 0.15)	0.01
Male recipient (yes *versus* non)	1.26	(−1.77; 4.28)	0.42	1.84	(0.05; 3.63)	0.04
Recipient BMI (kg/m²)	−0.42	(−0.64; −0.20)	<0.01			
Renal replacement therapy modality (ref: peritoneal dialysis)						
- Hemodialysis	5.18	(0.7; 9.65)	0.02	−4.09	(−6.72; −1.47)	<0.01
- Pre-emptive transplantation	−3.54	(−9.7; 2.62)	0.26	2.66	(−0.94; 6.26)	0.15
Time spent in dialysis (years)	0.35	(−0.01; 0.71)	0.06	−0.24	(−0.45; −0.03)	0.03
Anti-HLA class II antibodies (yes *versus* no)	6.48	(2.71; 10.25)	<0.01	−5.08	(−7.32; −2.84)	<0.01
Donor age (years)	−0.57	(−0.67; −0.48)	<0.01			
Donor BMI (kg/m²)	−0.21	(−0.47; 0.06)	0.13	0.21	(0.05; 0.37)	0.01
Donor vital status (ref: living donor)						
- Non cerebrovascular death	−3.20	(−6.78; 0.37)	0.08			
- Cerebrovascular death	−4.34	(−7.97; −0.72)	0.02			
- Donor after cardiac death	−11.76	(−17.69; −5.83)	<0.01			

Time is expressed as a continuous variable in years. Squared root time is included to account for a changing effect of time. Also, estimated GFR evolution over time is for square root time unit. Abbreviations: BMI = Body Mass Index, CI95% = Confidence interval 95%,.

**Table 4:** Multivariate Cox model for biopsy proven acute rejection.

	HR	CI95%	*p*-Value
CYP3A5 *1/- (versus CYP3A5 *3/*3)	1.01	(0.68; 1.49)	0.97
Male donor (yes *versus* no)	0.64	(0.47; 0.86)	<0.01
HLA-A-B-DR incompatibilities > 4 (yes *versus* no)	1.23	(0.87; 1.74)	0.24
Positive anti-HLA class II antibodies (yes *versus* no)	1.41	(1.00; 2.01)	0.05
Cold ischemia time (per 10 hours)	1.46	(1.19; 1.80)	<0.01

Abbreviations: HR = Hazard Ratio, CI95% = Confidence interval 95%, HLA = Human Leucocyte Antigen. 30 observations deleted due to missingness.

## Data Availability

The datasets used and analyzed during the current study are available from the corresponding author upon reasonable request.

## Supplementary Materials

The following are available online at https://www.mdpi.com/article/10.3390/jpm11101002/s1, Figure S1: Unadjusted curves of death censored graft survival using the Kaplan Meier estimator according to CYP3A5 genotype (*n* = 1114 patients), Table S1: Histological lesions on the last kidney biopsy before graft loss, according to CYP3A5 genotype, Table S2: Linear mixed model for Tacrolimus daily dose/body weight (mg/kg/day) according to CYP3A5 expression from 1 year post transplantation, Table S3: Linear mixed model for Tacrolimus C0 over time according to CYP3A5 genotype from 1 year post transplantation, Table S4: Linear mixed model for C0/Tacrolimus daily dose estimation over time according to CYP3A5 expression from 1 year post transplantation, Table S5: Multivariate Cox model for death censored graft survival.
