# Supplementary material for: Impact of Tacrolimus Daily Dose Limitation in Renal Transplant Recipients Expressing CYP3A5: A Retrospective Study
Source: J Pers Med. 2021 Oct 2;11(10):1002. doi: 10.3390/jpm11101002 (PMC8539387; doi:10.3390/jpm11101002)
Supplement: Supplementary file 1 [file jpm-11-01002-s001.zip › jpm-1338012-supplementary.pdf]

Journal of Personalized Medicine

Supplemental Table S1. Histological lesions on the last kidney biopsy before graft loss, according to CYP3A5 genotype.

	*CYP3A*3/*3* (n=74)	*CYP3A*3/-* (n=13)	p-value
Chronic ABMR	8 (10.8%)	1 (7.69%)	1.000
Acute ABMR	11 (14.9%)	1 (7.69%)	0.684
Acute TCMR	16 (21.6%)	1 (7.69%)	0.449
Chronic TCMR	3 (4.05%)	0	1.000
Borderline rejection	13 (17.6%)	0	0.202
Acute tubular necrosis	29 (39.2%)	6 (46,2%)	0.868
Pyelonephritis	4 (5.41%)	0	1.000
Recurence of primary kidney disease	4 (5.41%)	2 (15.4%)	0.218
Tacrolimus chronic nephrotoxicity	11 (14.9%)	3 (23.1%)	0.432
BK virus nephropathy	8 (10.8%)	0	0.600
Thrombotic microangiopathy	10 (13.5%)	5 (38.5%)	0.043
Donor specific lesions	34 (45.9%)	4 (30.8%)	0.475
De novo glomerulonephritis	0	2 (15.4%)	0.021

Abbreviations : ABMR = Antibody Mediated Rejection, TCMR = T cell Mediated Rejection

Supplemental Table S2. Linear mixed model for Tacrolimus daily dose / body weight (mg/kg/day) according to CYP3A5 expression from 1 year post transplantation.

	Association with Tac daily dose / body weight (baseline effect)			Association with Tac daily dose / body weight evolution (slope effect)		
	Coefficients	CI95%	p-value	Coefficients	CI95%	p-value
Referential value	0.0656	(0.0629; 0.0682)	< 0.01	- 0.0027	(- 0.0031; - 0.0025)	< 0.01
*CYP3A5 *1/-* (*versus CYP3A5* *3/*3)	0.0342	(0.0280; 0.0404)	< 0.01	- 0.0006	(-0.0015; 0.0003)	0.17

Time is expressed as a continuous variable in years.
The mean Tacrolimus daily dose / body weight (mg/kg/day) for the estimated model is calculated as: Tacrolimus daily dose / body weight = 0.0656 + 0.0342*[*CYP3A5 *1/-*] + (– 0.0027 – 0.0006*[*CYP3A5 *1/-*])*time (in years) with 1 if *CYP3A5 *1/-* and 0 otherwise.
Abbreviations: CI95% = Confidence Interval 95%; Tac = Tacrolimus

Supplemental Table S3. Linear mixed model for Tacrolimus C0 over time according to *CYP3A5* genotype from 1 year post transplantation.

	Association with Tacrolimus C0 (baseline effect)			Association with Tacrolimus C0 evolution (slope effect)		
	Coefficients	CI95%	p-value	Coefficients	CI95%	p-value
Referential value	8.36	(8.07; 8.65)	< 0.01	-1.18	(-1.32; -1.03)	< 0.01
*CYP3A5 *1/-* (*versus CYP3A5* *3/*3)	-1.36	(-2.03 ; -0.69)	< 0.01	0.13	(-0.21 ; 0.48)	0.46

Time is expressed as a continuous variable in years. Square root time is included to account for a changing effect on time. Thus, estimated Tacrolimus C0 evolution over time is for square root time unit. The mean Tacrolimus daily dose / body weight (mg/kg/day) for the estimated model is calculated as: Tacrolimus daily dose / body weight = 8.36 – 1.36*[*CYP3A5 *1/-*] + (– 1.18+0.13*[*CYP3A5 *1/-*])*sqr (time) with 1 if *CYP3A5 *1/-* and 0 otherwise.
Abbreviations: C0 = Tacrolimus blood through concentration, CI95% = Confidence Interval 95%; Tac = Tacrolimus

Supplemental Table S4. Linear mixed model for C0 / Tacrolimus daily dose estimation over time according to CYP3A5 expression from 1 year post transplantation.

	Association with the C0 / daily dose (baseline effect)			Association with the C0 / daily dose evolution (slope effect)		
	Coefficients	CI95%	p-value	Coefficients	CI95%	p-value
Referential value	2.00	(1.90; 2.09)	< 0.01	-0.01	(-0.03; 0.01)	0.22
*CYP3A5 *1/-* (*versus CYP3A5* *3/*3)	- 1.01	(- 1.23; - 0.78)	< 0.01	0.01	(- 0.04; 0.05)	0.81

Time is expressed as a continuous variable in years. The mean C0 / daily dose for the estimated model is calculated as: C0/daily dose(time) = 2.00 – 1.01*[*CYP3A5 *1/-*] + (-0.01 + 0.01*[*CYP3A5 *1/-*])*time with 1 if *CYP3A5 *1/-* and 0 otherwise. Abbreviations: C0 = Tacrolimus blood through concentration, CI95% = Confidence Interval 95%.

Supplemental Table S5. Multivariate Cox model for death censored graft survival.

	HR	CI95%	p-value
*CYP3A5 *1/-* (*versus CYP3A5 *3/*3*)	0.73	[0.43; 1.23]	0.23
Recipient age (per 10 years)	0.99	[0.80; 1.21]	0.89
Donor age (per 10 years)	1.14	[0.94; 1.38]	0.17
Retransplantation (yes *versus* no)	0.99	[0.58; 1.67]	0.96
Time spent in dialysis (per 1 year)	1.05	[1.02; 1.08]	< 0.01
Donor vital status			
- Living donor	Ref.		
- Non cerebrovascular donor death	3.62	[0.80; 16.38]	0.09
- Cerebrovascular donor death	5.36	[1.18; 24.23]	0.03
- Donor after cardiac death	12.56	[2.50; 63.13]	< 0.01
Cold ischemia time (per 10 hours)	1.06	[0.79; 1.44]	0.68
Occurrence of BPAR (yes *versus* no)	4.18	[2.81; 6.22]	< 0.01

Abbreviations: HR = Hazard Ratio, CI95% = Confidence interval 95%, BPAR = Biopsy Proven Acute Rejection. Occurrence of BPAR was a time dependent covariate. 22 observations were deleted due to missingness

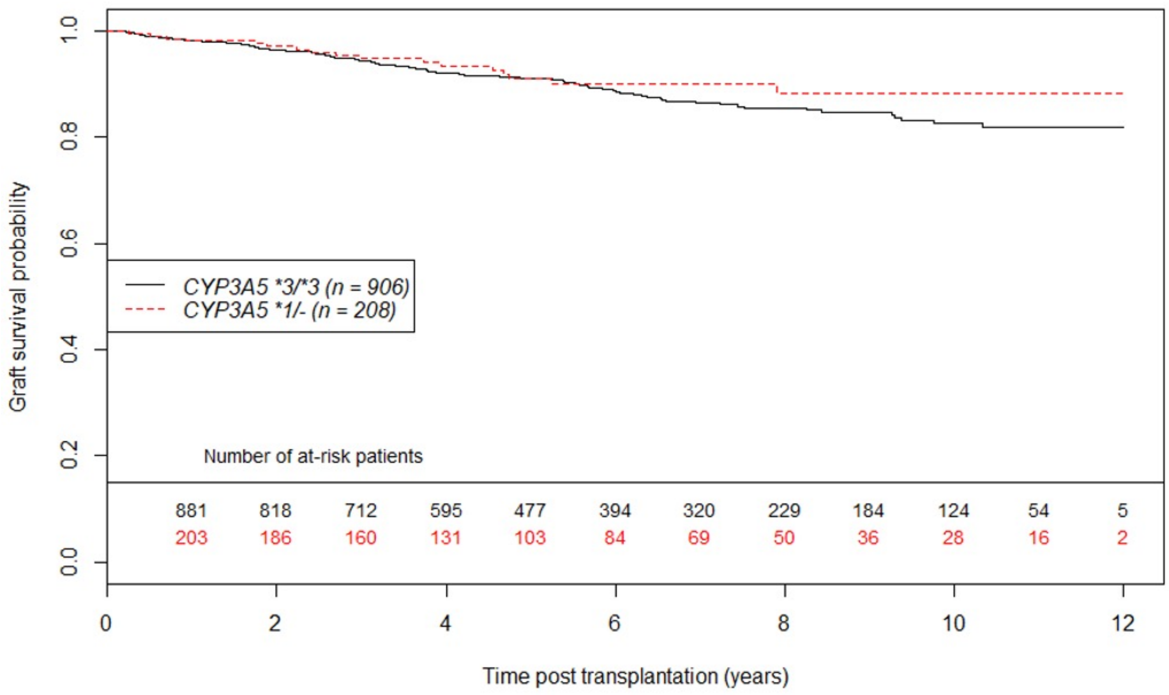

Supplemental Figure S1: Unadjusted curves of death censored graft survival using the Kaplan Meier estimator according to CYP3A5 genotype (n = 1114 patients).
